# The Status of Selenium and Zinc in the Urine of Children From Endemic Areas of Kashin-Beck Disease Over Three Consecutive Years

**DOI:** 10.3389/fnut.2022.862639

**Published:** 2022-04-08

**Authors:** Xin Kang, Yanli Liu, Yi Gong, Lin Huang, Hongliang Liu, Minhan Hu, Ruitian Huang, Feihong Chen, Sijie Chen, Feiyu Zhang, Yujie Ning, Cheng Li, Rong Zhou, Hongmou Zhao, Xi Wang, Xiong Guo

**Affiliations:** ^1^Department of Sports Medicine, Honghui Hospital of Xi'an Jiaotong University, Xi'an, China; ^2^Department of Occupational and Environmental Health, School of Public Health, Xi'an Jiaotong University Health Science Center, Xi'an, China; ^3^Department of Orthopedics, Honghui Hospital of Xi'an Jiaotong University, Xi'an, China; ^4^Key Laboratory of Trace Elements and Endemic Diseases, Collaborative Innovation Center of Endemic Disease and Health Promotion for Silk Road Region, School of Public Health, Xi'an Jiaotong University Health Science Center, Xi'an, China; ^5^Department of Kashin-Beck Disease and Keshan Disease Prevention, Shaanxi Provincial Institute for Endemic Disease Control, Xi'an, China; ^6^Foot and Ankle Surgery Department, Honghui Hospital of Xi'an Jiaotong University, Xi'an, China

**Keywords:** selenium/zinc content, Kashin-Beck disease, children, urine, hair

## Abstract

Selenium deficiency is one of the main risk factors for Kashin-Beck disease (KBD). This study aimed to detect the status of selenium and zinc in the urine of children from endemic areas of KBD over three consecutive years and to evaluate whether selenium and zinc levels in children in Shaanxi Province remain normal after stopping selenium supplementation. The samples of urine were collected in consecutive years (2017–2019) to detect selenium content by hydride generation atomic fluorescence spectrometry (HGAFS) and to detect zinc content by atomic absorption spectrophotometry (AAS). Generalized estimation equation (GEE) analysis was integrated to assess the comprehensive nutritional status and dietary structure of children. Data were processed in duplicate and analyzed by SPSS 18.0. This study included 30 X-ray-positive KBD cases and 123 healthy children aged 7–12 years. A total of 424 urine and 137 hair samples were collected over three consecutive years for selenium determination. The mean value of urinary selenium in all subjects was 6.86 μg/l (2017), 8.26 μg/l (2018), and 4.04 μg/l (2019), and the mean value of urinary zinc in all subjects was 0.36 mg/l (2017), 0.39 mg/l (2018), and 0.31 mg/l (2019) for the three consecutive years of 2017–2019. The mean values of urinary selenium were 6.56 and 6.94 μg/l (2017), 8.69 and 8.14 μg/l (2018), and 4.57 and 3.90 μg/l (2019) in the KBD-X and normal groups, respectively; and the mean value of urinary zinc were 0.38 and 0.35 mg/l (2017), 0.41 and 0.39 mg/l (2018), and 0.43 and 0.28 mg/l (2019) in the KBD-X and normal groups, respectively. The mean value of hair selenium in 137 subjects was 275.08 μg/kg and the mean values of hair selenium were 267.48 and 276.61 μg/kg in the KBD-X group and normal group, respectively. The level of selenium/zinc showed a trend of increasing first and then decreasing during the three consecutive years. The level of selenium in all subjects from the endemic areas was lower than normal, which reminds us to monitor the state of KBD constantly and adjust selenium salt supplementation in accordance with the changes in the KBD state.

## Introduction

Kashin-Beck disease (KBD) is a chronic, symmetrical, deforming, regionally distributed osteoarthritic disease with the main pathological changes including chondrocyte necrosis and extracellular matrix degeneration. The clinical manifestations mainly include shortened and enlarged fingers, deformed limb joints, and even dwarfism in some advanced patients (1–3). KBD mainly occurs in children aged 3–12 years and invades the epiphyseal cartilage and epiphyseal plate cartilage, which seriously affects their growth and development (4). According to the China Health Statistical Yearbook 2020 (http://www.nhfpc.gov.cn), there are more than 17,000 patients and more than 100 million residents at risk. The etiology and pathology of KBD are still unclear, and the biogeochemical hypothesis suggests that KBD is caused by a significantly lower or excess level of several elements (5).

Trace elements, such as selenium and zinc, are important components of many enzymes in organisms and are involved in many key metabolic processes *in vivo* (6). Previous studies have suggested that environmental Se deficiency is the main risk factor for KBD (7), and the supplementation of Se and Zn has been verified to be effective in preventing and repairing KBD (8, 9). Some researchers found that there was no significant difference in the content of Zn^2+^ in the hair of adolescent patients with KBD and the normal control group, but it was significantly higher in the serum of adolescent patients with KBD than that of the normal control group (9). In addition, it was found that the hair Zn^2+^ content of children aged 7–12 in a KBD endemic area in 1995 was significantly higher than that of children in the same cohort in 1985 in a prospective study (10). In recent years, the incidence of KBD has declined in non-Se/Zn supplemented KBD areas, and only a few new cases have been detected. The reasons for the decrease in the incidence of KBD are unknown. However, after stopping selenium salt supplementation from 1 July 2012 (11), learning whether the selenium and zinc *in vivo* nutritional status of children in endemic areas can reach and maintain the level of non-endemic areas is crucial to eliminating KBD.

In recent years, emerging studies have found that a balanced diet, such as the regular consumption of meat and eggs, which could provide various trace elements needed by the body, may play an important role in preventing KBD. With the improvement of social and economic levels in the endemic areas of Shaanxi Province, the current comprehensive nutritional status-trace elements may be maintained at normal levels. Therefore, in this study, we detected the status of selenium and zinc in the urine of children from endemic areas of KBD in three consecutive years to determine selenium and zinc levels in children from six endemic counties and to evaluate whether selenium and zinc levels in children from Shaanxi Province remain normal after stopping selenium supplementation.

## Methods and Materials

### Study Design and Population

**Figure 1:**
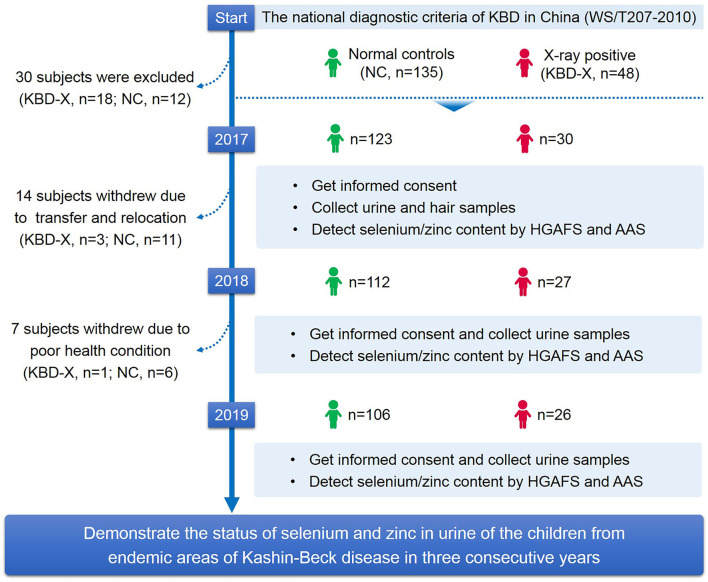
Study design. This flow chart shows the selection process of subjects and the main methods and technologies used in this study.

A total of 48 X-ray-positive KBD cases (KBD-X) and 135 normal controls (NC) from Shaanxi Province, an endemic area of KBD, were recruited at the beginning of this study. The stratified random sampling method was used for collecting subjects from Yongshou, Xunyi, Chunhua, Linyou, Qianyang, and Long counties, six of the endemic areas for KBD in Shaanxi Province in China. The national diagnostic criterion of KBD in China (WS/T207-2010) was used to diagnose the patients. Healthy controls without clinical symptoms of KBD were collected and matched with patients with KBD by sex and age. Subjects were excluded when they had other types of osteoarthropathy and other chronic diseases, such as cardiovascular disease, diabetes, and hypertension. According to the inclusion and exclusion criteria of the sample selection described above, 30 KBD-X and 123 NC were finally recruited into a 3-year cohort in the study (Table 1). In this study, there was one follow-up time point in year one, followed by one each year thereafter, including a comprehensive collection of biospecimens and data. The selection process of subjects is shown in detail in the research design (Figure 1).

**Table 1:** Characteristics of 153 children participants in this study.

**Variables**	**X-ray positive KBD** **(*n* = 30)**	**Children Controls** **(*n* = 123)**	***P***
Age (years)	9.2 ± 1.8	9.0 ± 1.0	0.669
Male, n (%)	14	63	−
Female, n (%)	16	60	−
BMI	16.9 ± 1.7	22.1 ± 2.9	0.001
Yongshou County	5	19	−
Chunhua County	5	20	−
Xunyi County	5	22	−
Qianyang County	4	20	−
Long County	5	20	−
Linyou County	6	22	−

### Collection and Processing of Hair and Urine Samples

For hair samples, ~2 g of children's occipital hair was collected using clean stainless-steel scissors. For girls with long hair, the root was cut at ~1 cm, the tips were removed, and the proximal 3–5 cm of hair was retained. Boys with short hair were given a haircut and their occipital hair was collected. All the hair samples were wrapped in clean envelopes and stored at room temperature. Before determination, each hair sample was cut into 3–5 mm sections and placed in a 50 ml beaker. Then ~20 ml of neutral detergent dissolved in distilled water was added and the samples were soaked for 30 min and stirred every 5 min with a clean glass rod. Then the hair samples were rinsed with tap water, distilled water, and ultrapure water in turns, and put in a clean filter paper bag to dry at 60°C for further analysis. For urine samples, the middle urine samples were collected after an overnight fast and immediately centrifuged at 1,500 rpm for 10 min to separate impurities. The supernatants were placed in sterile conical tubes and immediately frozen at −80°C for further analysis.

### Detection of Selenium/Zinc Content in Samples

All samples were digested using HNO_3_, HClO_4_ mixed digestion solution (4:1), and then potassium borohydride was added to 160°C graphite-controlled temperature electric digester for digestion until the solution was a white smoke of <1 ml (if the solution became black, a small amount of HNO_3_ was added until the solution in the tube was colorless or slightly yellow). Then, 1:1 hydrochloric acid (4 ml) was added while the solution was hot, heated for 8 min, removed, and allowed to cool down. The volume was fixed to 15 ml with distilled water, mixed well, and measured after 30 min. Selenium contents in all samples were determined in triplicate by hydride generation atomic fluorescence spectrometry (HGAFS) (AFS-9750, Haiguang Co., Ltd., Beijing), selenium standard solution: 1,000 μg/ml in accordance with GSB 04-1751-2004 Guobiao Testing & Certification Co., Ltd. Zinc contents in all samples were determined in triplicate by atomic absorption spectrophotometry (AAS) (DUO240, Agilent Co., Ltd., USA), zinc standard solution: 1,000 μg/ml in accordance with GSB 04-1761-2004 Guobiao Testing & Certification Co., Ltd.). Selenium standard curve: 0.2, 0.4, 0.6, 0.8, 1.0 μg/ml; zinc standard curve: 0.04, 0.08, 0.12, 0.16, 0.2 μg/ml.

### Quality Control

All subjects recruited as KBD-X and healthy controls in this study were enrolled according to the national diagnostic criteria of KBD [WS/T 207-2010] and general clinical examination. Preinvestigation was performed in Yongshou and Xunyi Counties to improve and adjust the questionnaire and study design. All investigators had a professional background in public health and were uniformly trained before the study. To ensure the accuracy and reliability of the test results, blank controls and reference materials (Chinese national-level standards GBW09101 for hair and urine samples) were set for each determination. Two aliquots of each sample were detected in triplicate, the average values were used for analysis, and a recovery test was performed for every 20 samples. In addition, raw data were independently entered in duplicate.

### Statistical Analyses

The data were statistically analyzed using SPSS 18.0 software. All repeated measurements had outliers removed by Grubb's test. The results for continuous variables are expressed as the mean ± standard deviation ($\bar{\text{X}}$ ± SD). Normality tests were performed before comparisons and correlation analysis. Non-parametric tests were used when the data were abnormally distributed. Generalized estimating equations (GEE) allow for the assessment of change between baseline and follow-up from all wave pairs in a single analysis while statistically controlling for interdependence among observations contributed by the same individuals (12–14). GEE analysis was used to identify factors affecting Se content. A *p* < 0.05 suggests a significant difference.

The purpose of the investigation was explained to the subjects and their guardians before filling the questionnaire and sampling to gain informed consent and cooperation. This study was approved by the Ethics Committee of Xi'an Jiaotong University.

## Results

### General Information of the Subjects

A total of 153 participants were enrolled from KBD endemic areas, Yongshou, Xunyi, Chunhua, Linyou, Qianyang, and Long counties in Shaanxi Province in China. Due to the low level of KBD morbidity in Shaanxi Province in recent years, only 30 KBD-X cases and 123 NC were recruited for 3 consecutive years in our study. Detailed information on all participants is presented in Table 1. In addition, 14 subjects (KBD-X, *n* = 3; normal control, *n* = 11) were lost to follow-up in the second year due to transfer to another school or relocation, and seven subjects (KBD-X, *n* = 1; normal controls, *n* = 6) were lost to follow-up in the third year due to poor health conditions.

### The Selenium Content in Urine From the KBD X-Ray-Positive Group and Normal Group Over Three Consecutive Years

**Figure 2:**
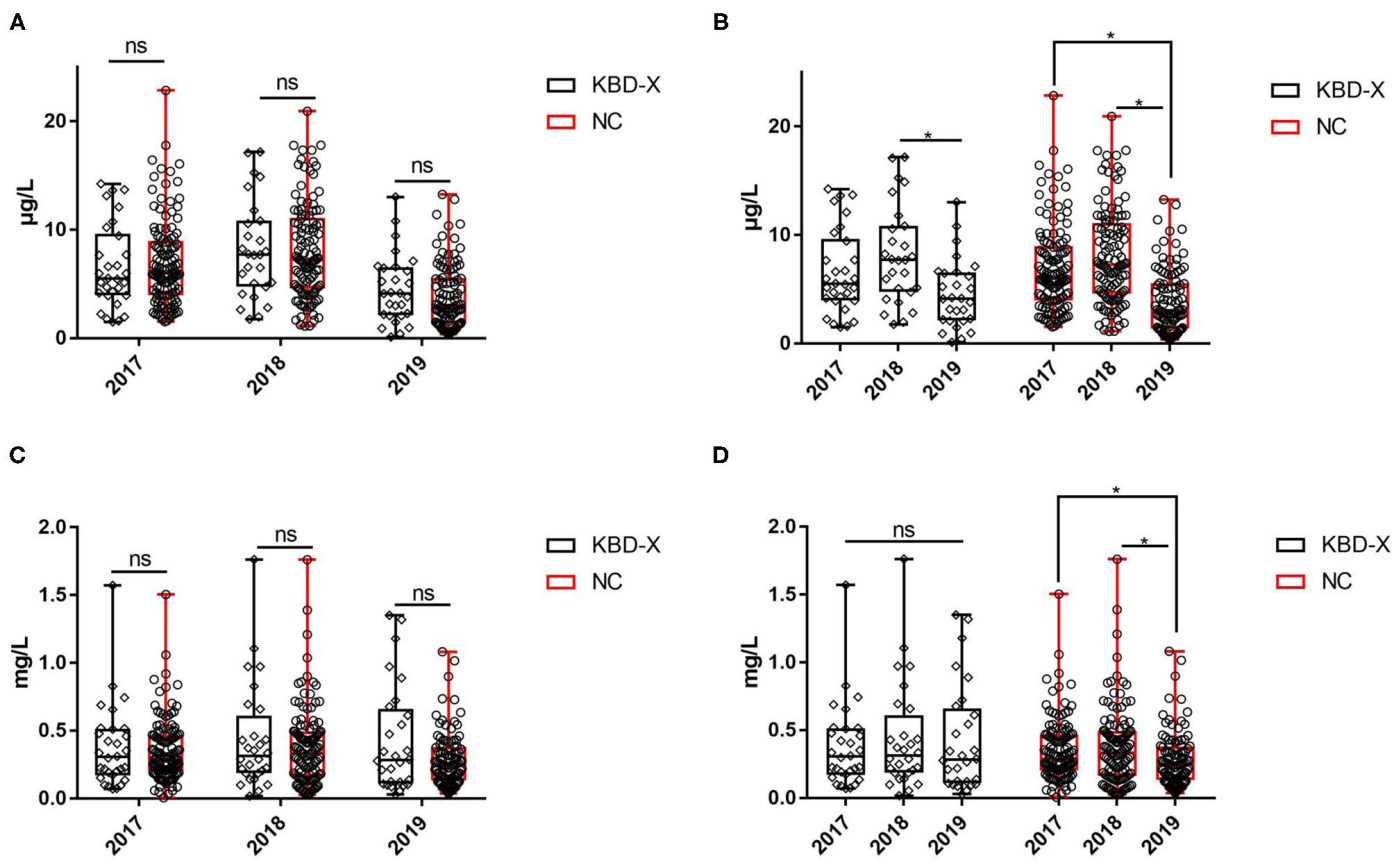
The content of urinary selenium and zinc between the KBD-X group and NC group in three consecutive years. **(A,B)** The content of urinary selenium, **(C,D)** the content of urinary zinc.

**Figure 3:**
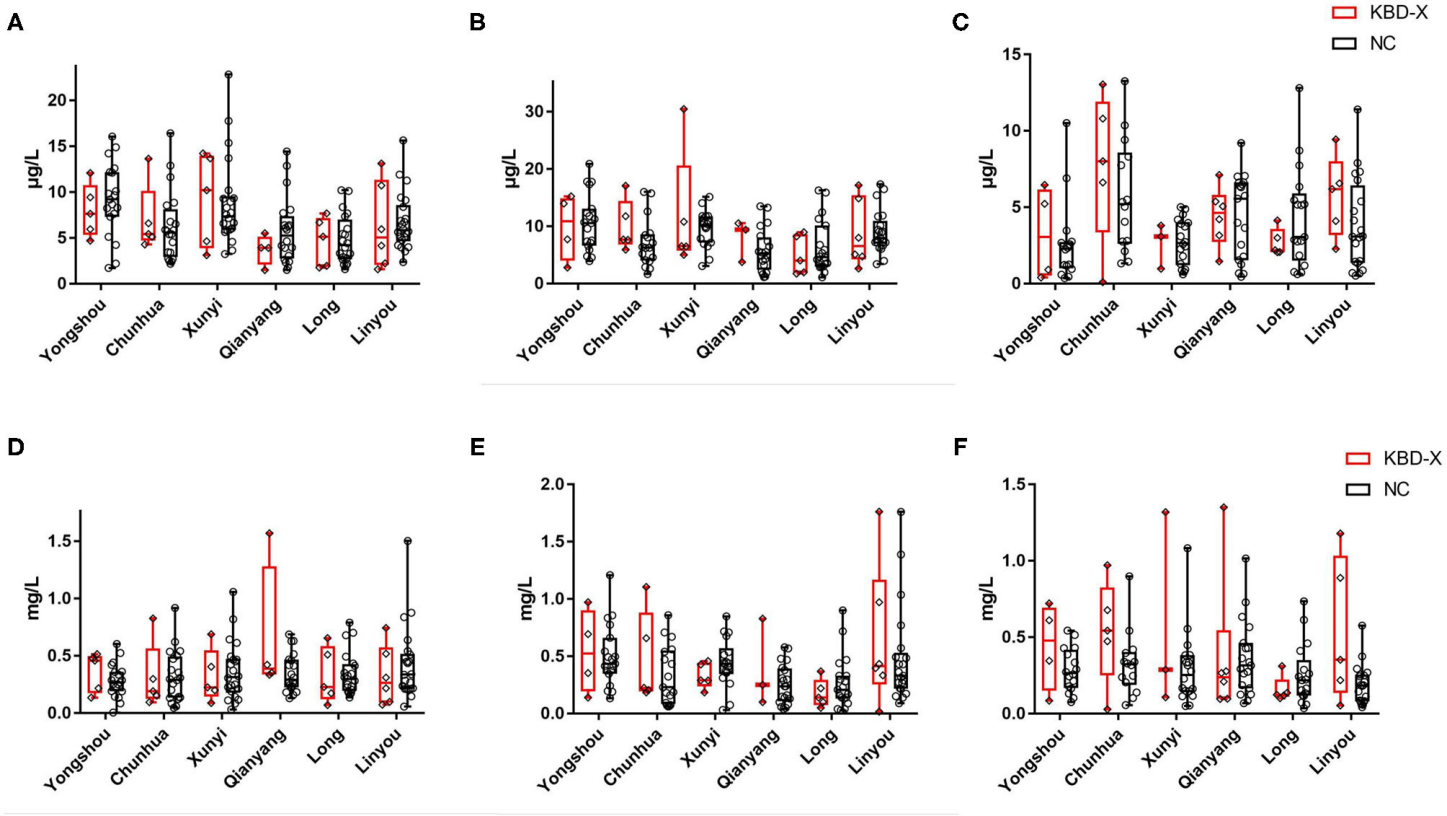
The content of urinary selenium and zinc in the KBD-X and NC groups in six (Yongshou, Chuanhua, Xunyi, Qianyang, Long, and Linyou) counties. **(A)** The content of urinary selenium in 2017, **(B)** the content of urinary selenium in 2018, **(C)** the content of urinary selenium in 2019, **(D)** the content of urinary zinc in 2017, **(E)** the content of urinary zinc in 2018, and **(F)** the content of urinary zinc in 2019.

**Figure 4:**
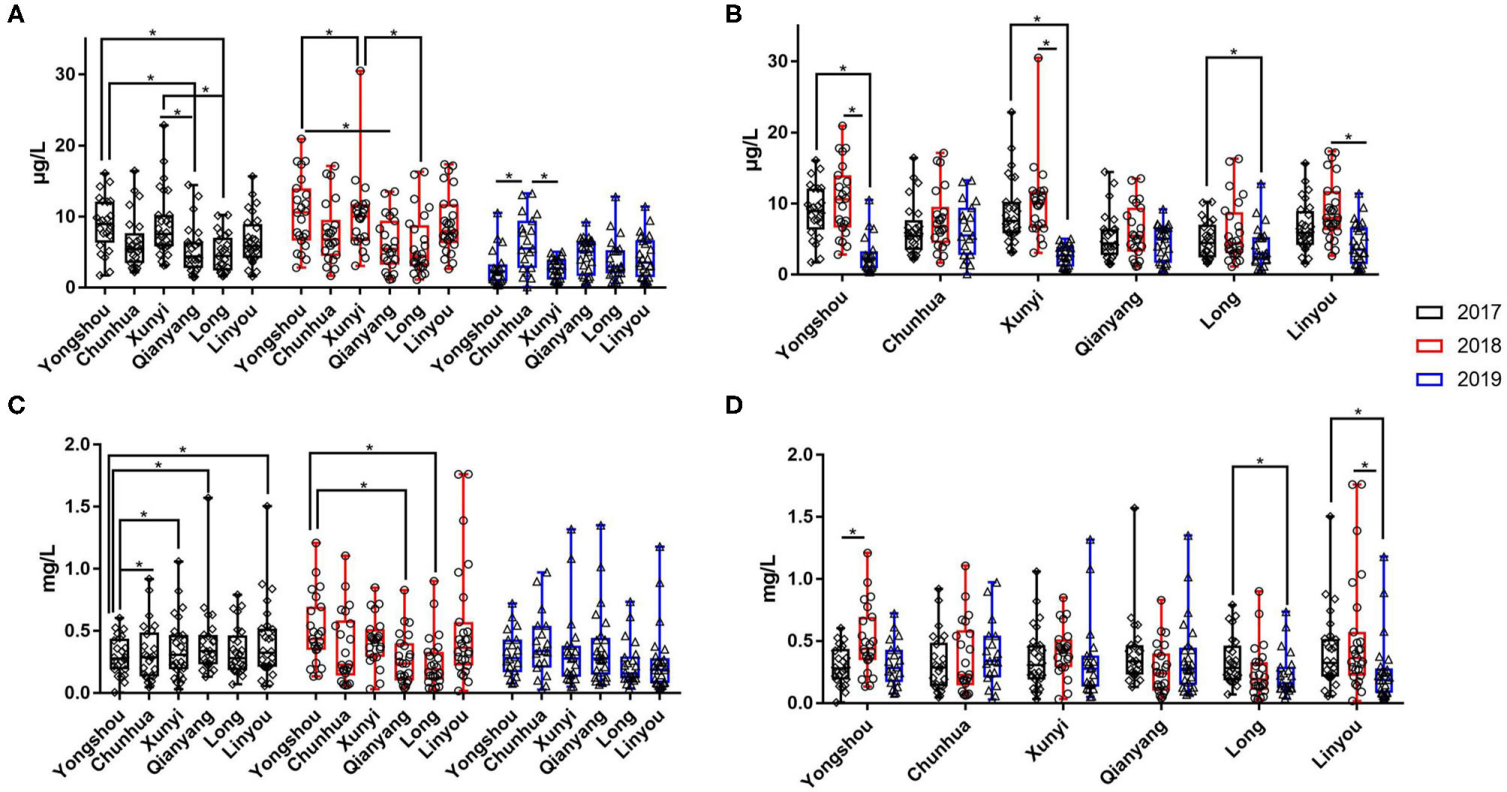
The content of urinary selenium and zinc of all subjects in six (Yongshou, Chuanhua, Xunyi, Qianyang, Long, and Linyou) counties in three consecutive years. **(A,B)** The content of urinary selenium, **(C,D)** the content of urinary zinc. *P < 0.05.

Over three consecutive years (2017–2019), the mean value of urinary selenium in all subjects was 6.86 μg/l (2017), 8.26 μg/l (2018), and 4.04 μg/l (2019), and the mean values of urinary selenium were 6.56 and 6.94 μg/l (2017), 8.69 and 8.14 μg/l (2018), and 4.57 and 3.90 μg/l (2019) in the KBD-X and normal groups, respectively (Figure 2; Table 2). There was no significant difference in the urinary selenium content between theKBD-X and NC group (Figure 2A); however, there were significant differences in the urinary selenium content between 2018 and 2019 in the KBD-X group, and between 2017 and 2019 and between 2018 and 2019 in the NC group (Figure 2B). There was no significant difference among sexes in the six different KBD counties by Fisher's exact probability test. In addition, the mean value of urinary selenium in different counties in 2017 were 8.94 μg/l in Yongshou county (7.98 μg/l in KBD-X, 9.19 μg/l in NC, Figure 3A), 6.37 μg/l in Chunhua county (7.02 μg/l in KBD-X, 6.21 μg/l in NC, Figure 3A), 8.83 μg/l in Xunyi county (9.19 μg/l in KBD-X, 8.75 μg/l in NC, Figure 3A), 5.45 μg/l in Qianyang county (3.73 μg/l in KBD-X, 5.79 μg/l in NC, Figure 3A), 4.80 μg/l in Long county (4.66 μg/l in KBD-X, 4.84 μg/l in NC, Figure 3A), and 6.68 μg/l in Linyou county (6.31 μg/l in KBD-X, 6.78 μg/l in NC, Figure 3A; Table 3). There were significant differences between Long and Xunyi counties, Long and Yongshou counties, Qianyang and Xunyi counties, and Qianyang and Yongshou counties (Figure 3A). The mean value of urinary selenium in different counties in 2018 were 10.48 μg/l in Yongshou county (9.95 μg/l in KBD-X, 10.58 μg/l in NC, Figure 3B), 7.77 μg/l in Chunhua county (10.04 μg/l in KBD-X, 7.11 μg/l in NC, Figure 3B), 10.12 μg/l in Xunyi county (11.88 μg/l in KBD-X, 9.62 μg/l in NC, Figure 3B), 6.07 μg/l in Qianyang county (7.91 μg/l in KBD-X, 5.79 μg/l in NC, Figure 3B), 6.05 μg/l in Long county (5.00 μg/l in KBD-X, 6.32 μg/l in NC, Figure 3B), and 9.01 μg/l in Linyou county (8.76 μg/l in KBD-X, 9.08 μg/l in NC, Figure 3B) (Table 3). There were significant differences between Long and Xunyi counties, Long and Yongshou counties, and Qianyang and Yongshou counties (Figure 4A). The mean value of urinary selenium in different counties in 2019 were 2.75 μg/l in Yongshou county (3.25 μg/l in KBD-X, 2.62 μg/l in NC, Figure 3C), 6.22 μg/l in Chunhua county (7.72 μg/l in KBD-X, 5.68 μg/l in NC, Figure 3C), 2.75 μg/l in Xunyi county (2.62 μg/l in KBD-X, 2.76 μg/l in NC, Figure 3C), 4.41 μg/l in Qianyang county (4.40 μg/l in KBD-X, 4.40 μg/l in NC, Figure 3C), 3.95 μg/l in Long county (2.69 μg/l in KBD-X, 4.31 μg/l in NC, Figure 3C), and 4.24 μg/l in Linyou county (5.72 μg/l in KBD-X, 3.86 μg/l in NC, Figure 3C). There were significant differences between Yongshou and Chunhua counties, and Xunyi and Chunhua counties (Figure 4A) (Table 3). In addition, the comparison analysis of the urinary selenium content in each county among three consecutive years is shown in Figure 4B.

**Table 2:** The mean value of urinary selenium and zinc in KBD-X and normal controls in three consecutive years.

**Years**	**groups**	**Se (μg/L)** **x ±SD**	***P***	**Zn (mg/L)** **x ±SD**	***P***
2017	KBD-X NC	6.56 ± 3.82 6.94 ± 3.96	0.458	0.38 ± 0.30 0.35 ± 0.22	0.573
2018	KBD-X NC	8.69 ± 4.37 8.14 ± 4.39	0.976	0.41 ± 0.38 0.39 ± 0.28	0.628
2019	KBD-X NC	4.57 ± 3.12 3.90 ± 2.93	0.268	0.43 ± 0.39 0.28 ± 0.20	0.308
2017	−	6.86 ± 3.96		0.36 ± 0.24	
2018	−	8.26 ± 4.79		0.39 ± 0.31	
2019	−	4.04 ± 3.00		0.31 ± 0.26	

**Table 3:** The mean value of urinary selenium and zinc in KBD-X and normal controls in different counties.

**Years**	**Counties**	**groups**	**Se (μg/L)** **x ±SD**	**Average Se (μg/L)** x ±SD^*^	**Zn (mg/L) x ±SD**	**Average** **Zn (mg/L)** x ±SD^*^
2017	Yongshou Chunhua Xunyi Qianyang Long Linyou	KBD-X NC KBD-X NC KBD-X NC KBD-X NC KBD-X NC KBD-X NC	7.98 ± 2.90 9.19 ± 4.00 7.02 ± 3.79 6.21 ± 3.83 9.19 ± 5.09 8.75 ± 4.78 3.73 ± 1.66 5.79 ± 3.61 4.66 ± 2.70 4.84 ± 2.68 6.31 ± 4.68 6.78 ± 3.13	8.94 ± 3.78 6.37 ± 3.76 8.83 ± 4.75 5.45 ± 3.43 4.80 ± 2.63 6.68 ± 3.43	0.36 ± 0.17 0.28 ± 0.14 0.31 ± 0.29 0.32 ± 0.22 0.32 ± 0.23 0.37 ± 0.25 0.67 ± 0.60 0.35 ± 0.16 0.33 ± 0.24 0.34 ± 0.19 0.33 ± 0.26 0.43 ± 0.32	0.30 ± 0.15 0.32 ± 0.23 0.36 ± 0.25 0.40 ± 0.29 0.34 ± 0.20 0.41 ± 0.31
2018	Yongshou Chunhua Xunyi Qianyang Long Linyou	KBD-X NC KBD-X NC KBD-X NC KBD-X NC KBD-X NC KBD-X NC	9.95 ± 5.76 10.58 ± 5.00 10.04 ± 4.49 7.11 ± 4.25 11.88 ± 10.62 9.62 ± 3.15 7.91 ± 3.63 5.79 ± 3.76 5.00 ± 3.42 6.32 ± 4.62 8.76 ± 5.92 9.08 ± 3.72	10.48 ± 5.01 7.77 ± 4.38 10.12 ± 5.40 6.07 ± 3.74 6.05 ± 4.36 9.01 ± 4.18	0.54 ± 0.36 0.49 ± 0.26 0.47 ± 0.40 0.33 ± 0.25 0.33 ± 0.11 0.44 ± 0.21 0.39 ± 0.38 0.25 ± 0.17 0.17 ± 0.12 0.27 ± 0.23 0.65 ± 0.62 0.48 ± 0.42	0.51 ± 0.28 0.37 ± 0.29 0.42 ± 0.20 0.28 ± 0.20 0.25 ± 0.21 0.52 ± 0.47
2019	Yongshou Chunhua Xunyi Qianyang Long Linyou	KBD-X NC KBD-X NC KBD-X NC KBD-X NC KBD-X NC KBD-X NC	3.25 ± 3.04 2.62 ± 2.63 7.72 ± 4.92 5.68 ± 3.66 2.62 ± 1.47 2.76 ± 1.48 4.40 ± 1.94 4.40 ± 2.73 2.69 ± 0.90 4.31 ± 3.29 5.72 ± 2.70 3.86 ± 2.98	2.75 ± 2.65 6.22 ± 3.99 2.75 ± 1.45 4.41 ± 2.53 3.95 ± 2.99 4.24 ± 2.98	0.44 ± 0.28 0.28 ± 0.14 0.53 ± 0.34 0.32 ± 0.21 0.57 ± 0.65 0.29 ± 0.24 0.38 ± 0.48 0.36 ± 0.24 0.15 ± 0.08 0.25 ± 0.19 0.53 ± 0.47 0.18 ± 0.13	0.32 ± 0.18 0.38 ± 0.26 0.33 ± 0.32 0.37 ± 0.30 0.23 ± 0.18 0.26 ± 0.27

* *Showed Average selenium/zinc content of different counties*.

### The Content of Zinc in Urine From the KBD X-Ray-Positive Group and Normal Group Over Three Consecutive Years

For three consecutive years (2017–2019), the mean value of urinary zinc in all subjects was 0.36 mg/l (2017), 0.39 mg/l (2018), and 0.31 mg/l (2019), and the mean values of urinary zinc were 0.38 and 0.35 mg/l (2017), 0.41 and 0.39 mg/l (2018), and 0.43 and 0.28 mg/l (2019) in KBD-X group and normal group, respectively (Figure 2; Table 2). There was no significant difference between the KBD-X and normal groups (Figure 2C). However, there were significant difference in the urinary zinc content between 2017 and 2019, and between 2018 and 2019 in the NC group (Figure 2D). The mean value of urinary zinc in different counties in 2017 were 0.30 mg/l in Yongshou county (0.36 mg/l in KBD-X, 0.28 mg/l in NC, Figure 3D), 0.32 mg/l in Chunhua county (0.31 mg/l in KBD-X, 0.32 mg/l in NC, Figure 3D), 0.36 mg/l in Xunyi county (0.32 mg/l in KBD-X, 0.37 mg/l in NC, Figure 3D), 0.40 mg/l in Qianyang county (0.67 mg/l in KBD-X, 0.35 mg/l in NC, Figure 3D), 0.34 mg/l in Long county (0.33 mg/l in KBD-X, 0.34 mg/l in NC, Figure 3D), and 0.41mg/l in Linyou county (0.33 mg/l in KBD-X, 0.43 mg/l in NC, Figure 3D) (Table 3). There were significant differences between Yongshou and Xunyi counties, Yongshou and Chunhua counties, Yongshou and Linyou counties, and Yongshou and Qianyang counties (Figure 4C). The mean value of urinary zinc in different counties in 2018 were 0.51 mg/l in Yongshou county (0.54 mg/l in KBD-X, 0.49 mg/l in NC, Figure 3E), 0.37 mg/l in Chunhua county (0.47 mg/l in KBD-X, 0.33 mg/l in NC, Figure 3E), 0.42 mg/l in Xunyi county (0.33 mg/l in KBD-X, 0.44 mg/l in NC, Figure 3E), 0.28 mg/l in Qianyang county (0.39 mg/l in KBD-X, 0.25 mg/l in NC, Figure 3E), 0.25 mg/l in Long county (0.17 mg/l in KBD-X, 0.27 mg/l in NC, Figure 3E), and 0.52 mg/l in Linyou county (0.65 mg/l in KBD-X, 0.48 mg/l in NC, Figure 3E) (Table 3). There were significant differences between Long and Yongshou counties, and Qianyang and Yongshou counties. The mean value of urinary zinc in different counties in 2019 were 0.32 mg/l in Yongshou county (0.44 mg/l in KBD-X, 0.28 mg/l in NC, Figure 3F), 0.38 mg/l in Chunhua county (0.53 mg/l in KBD-X, 0.32 mg/l in NC, Figure 3F), 0.33 mg/l in Xunyi county (0.57 mg/l in KBD-X, 0.29 mg/l in NC, Figure 3F), 0.37 mg/l in Qianyang county (0.38 mg/l in KBD-X, 0.36 mg/l in NC, Figure 3F), 0.23 mg/l in Long county (0.15 mg/l in KBD-X, 0.25 mg/l in NC, Figure 3F), and 0.26 mg/l in Linyou county (0.53 mg/l in KBD-X, 0.18 mg/l in NC, Figure 3F) (Table 3). There were no significant differences between the six different counties (Figure 4C). In addition, the comparison analysis of the urinary zinc content in each county among three consecutive years is shown in Figure 4D.

### The Selenium/Zinc Content in Hair From the KBD X-Ray-Positive Group and Normal Group in 2017

**Figure 5:**
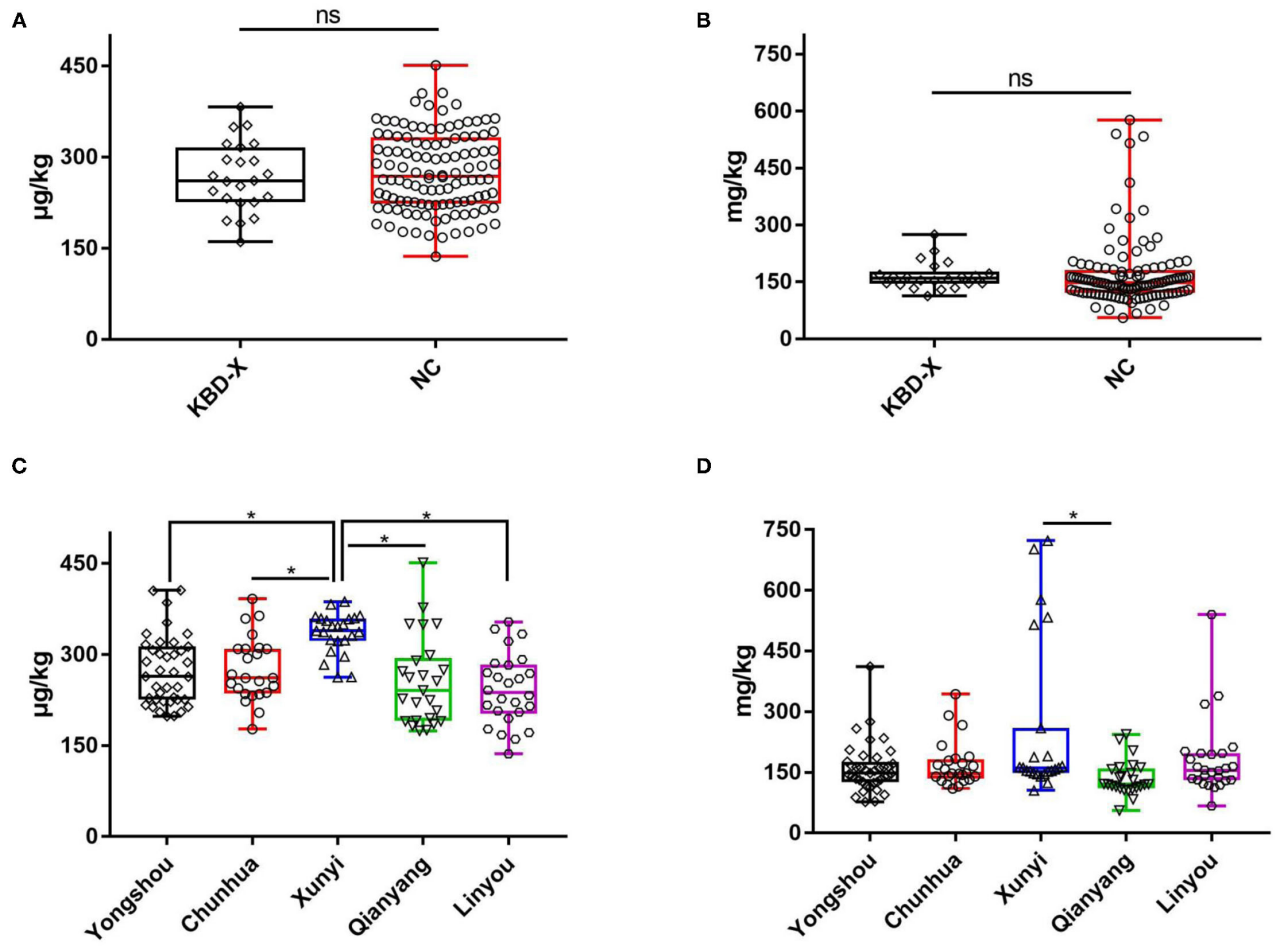
The content of selenium and zinc in hair in 2017. **(A)** The content of selenium in hair from KBD-X and NC groups, **(B)** the content of zinc in hair from KBD-X and NC groups, **(C)** the content of selenium in hair in five (Yongshou, Chuanhua, Xunyi, Qianyang, and Linyou) counties, and **(D)** the content of zinc in hair in five (Yongshou, Chuanhua, Xunyi, Qianyang, and Linyou) counties. *P < 0.05.

A total of 137 hair samples were collected in 2017. The mean value of hair selenium in 137 subjects was 275.08 μg/kg, and the mean values of hair selenium were 267.48 and 276.61 μg/kg in the KBD-X group and normal group, respectively. There was no significant difference between the two groups (Figure 5A). The mean values of hair selenium in different counties were 273.26 μg/kg in Yongshou County, 275.81 μg/kg in Chunhua County, 256.23 μg/kg in Qianyang County, 334.84 μg/kg in Xunyi County, and 242.40 μg/kg in Linyou County. There were significant differences between Xunyi and Yongshou counties, Xunyi and Chunhua counties, Xunyi and Linyou counties, and Xunyi and Qianyang counties (Figure 5C). The mean value of hair zinc in 137 subjects was 177.63 mg/kg, and the mean values of hair zinc were 190.01 and 175.13 mg/kg in the KBD-X group and normal group, respectively. There was no significant difference between the two groups (Figure 5B). The mean values of hair zinc in different counties were 160.34 mg/kg in Yongshou County, 169.90 mg/kg in Chunhua County, 136.05 mg/kg in Qianyang County, 257.49 mg/kg in Xunyi County, 180.05 mg/kg in Linyou County. There were significant differences between Xunyi and Qianyang counties (Figure 5D).

### GEE Analysis

In this study, the selenium/zinc content was detected three times in the same subjects over three consecutive years. The relationship between the content of selenium/zinc and KBD was analyzed by GEE, which considers the correlation between each measured value in different years and groups. GEE analysis showed that there were no significant differences in selenium between the groups (Wald χ^2^ = 0.172, *p* = 0.678), and the estimated marginal values were 6.640 in KBD-X and 6.409 in NC. There were significant differences in selenium within the times of detection (Wald χ^2^ = 41.593, *p* = 0.001), and the estimated marginal values were 6.927 in 2017, 8.176 in 2018, and 4.470 in 2019. GEE analysis showed that there were no significant differences in zinc between the groups (Wald χ^2^ = 2.330, *p* = 0.127), and the estimated marginal values were 0.400 in KBD-X and 0.344 in NC. There were significant differences in zinc within the times of detection (Wald χ^2^ = 5.744, *p* = 0.017), and the estimated marginal values were 0.373 in 2017, 0.407 in 2018, and 0.337 in 2019 (Table 4).

**Table 4:** The main effect of affecting factors on urine selenium and zinc contents in a study by GEE analysis.

**Factors**	**Se** **x ±SD**	**χ^2^**	***P***	**Zn** **x ±SD**	**χ^2^**	***P***
Groups
KBD-X Healthy controls Years	6.640 ± 0.51 6.409 ± 0.23	0.172	0.678	0.400 ± 0.034 0.343 ± 0.015	2.330	0.127
2017	6.927 ± 0.34	41.593	0.001	0.373 ± 0.021	5.744	0.017
2018	8.176 ± 0.43			0.407 ± 0.026		
2019	4.470 ± 0.29			0.337 ± 0.024		

## Discussion

In this study, we detected the status of selenium and zinc in urine samples collected from children who were continuously living in KBD-endemic areas of KBD over three consecutive years (2017, 2018, and 2019) to determine selenium and zinc levels in children from six endemic counties and to evaluate whether selenium and zinc levels in children in Shaanxi Province remained normal after stopping selenium supplementation from 2012. This study demonstrated that the level of zinc was maintained at a normal status, while the level of selenium was lower than the normal status. The levels of selenium and zinc showed a trend of increasing first and then decreasing during the three consecutive years.

It has been demonstrated that selenium deficiency is one of the crucial etiological factors for KBD, and the relevance between Se and KBD relies on a specific relationship between the morbidity of KBD and the content of Se in several samples, such as soil, drinking water, food, and human biological samples (15, 16). A previous study found that Se could improve cell aging and strengthen lipid peroxidation (17, 18), which played a key role in articular cartilage damage in patients with KBD (19). Moreover, Se supplementation has been suggested to be functional in preventing new cases of KBD in children based on meta-analysis (20). Hongzao et al. found that the status of selenium deficiency can lead to the thinning of rat medullary plate cartilage, fewer cells in each layer, decreased volume and number of hypertrophic cells, and other nutritional changes, which eventually lead to cartilage development disorders (21). A variety of selenoproteins could participate and play a key role in the growth and development of rat bone cartilage (22). A study detected some significantly downregulated selenium-deficiency response genes (postn, clec3b, Cyp8b1, fbln1, pygb, and SIGIRR) in chondrocytes from patients with KBD (23). In addition, an epidemiological study showed that KBD endemic areas are distributed in an oblique strip from northeast to southwest, which is roughly consistent with the distribution belt in low selenium areas in China (24). According to a meta-analysis on the selenium level in KBD and non-KBD endemic areas, the selenium content in the environment such as drinking water, soil, flour, and corn in KBD endemic areas was generally lower than that in non-KBD endemic areas. At the same time, the selenium content in the blood, urine, and hair of patients in KBD endemic areas was lower than that of normal controls (25). However, other countries, such as New Zealand and Finland, with low soil Se contents did not have any KBD cases, which could be caused by a substantially higher intake of Se. This may be because of the wide availability of non-local and imported food, or this may indicate that Se might not be the only risk factor for KBD (23). In 2012, Shaanxi Province decided to stop selenium salt supplementation due to the decrease in the incidence of KBD in children. This investigation found that the level of selenium in all subjects the from endemic areas was lower than normal, which reminds us to monitor the state of KBD constantly and make adjustments to stop selenium salt supplementation in line with changes in the state of KBD.

Zinc is closely related to the synthesis of enzymes, nucleic acids, and proteins and is an essential microelement for humans (26). A conundrum still exists in research on the relationship between Zn and KBD due to inconsistent results, but it has been found that Zn combined with Se can significantly help repair metaphyseal lesions in the articular cartilage of patients with KBD (8), indicating that Zn might play a vital role in the pathogenesis of KBD, although the mechanism is unknown. In a previous meta-analysis, the serum Zn level was lower in patients with KBD than in healthy controls, whereas the hair Zn level was higher in patients with KBD than in healthy controls (9). However, in this study, the level of zinc in all subjects from the endemic areas was within the normal status range. GEE analysis showed that the level of zinc showed a trend of increasing first and then decreasing during the three consecutive years. Nevertheless, based on the inconsistency of the results, the relationship between zinc and KBD requires further study.

Hair has been broadly used to estimate the relationship between nutritional elements and human health for years, while urine was recently considered a good indicator to reflect the level of trace elements *in vivo* (1, 2). In this study, we detected selenium and zinc levels in urine and hair samples of children in KBD-endemic areas in 2017. There were no significant differences in selenium and zinc levels in children's urine and hair between the KBD-X group and the normal control group. Among the six counties, children in Xunyi County had the higher selenium content both in hair and urine samples, while children in Qianyang County had the lower selenium content both in hair and urine samples. In addition, children in Xunyi County had the highest zinc content in hair, and children in Lingyou County had the highest zinc content in urine. Different geographic environments, dietary habits, and prevention strategies of KBD may affect the selenium and zinc contents in hair and urine. Dietary habits are an important factor affecting the selenium/zinc level in children's urine. Some primary schools in endemic areas have implemented the “egg-milk” project, which can ensure that children eat one egg and one packet of milk every day. In addition, children in different endemic areas usually have different habits in consuming fruits and snacks, which could also affect the content of trace elements in the body.

The sample size is limited in this study. Since the incidence of juvenile KBD has decreased dramatically and almost no new juvenile cases were diagnosed in Shaanxi Province recently, the sample size of the KBD-X group was limited. Although, the serum is a better indicator to reflect the level of trace elements *in vivo*, we chose urine instead of serum because it is difficult to collect children's blood due to the invasive sampling method.

## Conclusions

In summary, the status of selenium and zinc in the urine of children from endemic areas of KBD over three consecutive years was detected to determine selenium and zinc levels from Shaanxi endemic areas and to evaluate whether selenium and zinc levels in children in Shaanxi Province remain at normal levels and to observe the varying trends after stopping selenium supplementation.

## Data Availability Statement

The raw data supporting the conclusions of this article will be made available by the authors, without undue reservation.

## Ethics Statement

The studies involving human participants were reviewed and approved by the Human Ethics Committee of Xi'an Jiaotong University. Written informed consent to participate in this study was provided by the participants' legal guardian/next of kin.

## Author Contributions

XK, YL, HZ, and XW: conception and design. YG, XW, LH, HL, and CL: sample collection. LH, YL, YG, RZ, MH, SC, FZ, RH, FC, and XW: selenium determination and data analyses. XK, HZ, XW, YN, and XG: data interpretation and drafting manuscript. YN, HZ, XW, and XG: revising manuscript content. All authors contributed to the article and approved the submitted version.

## Funding

This study was financially supported by the National Natural Science Foundation of China (81803178 and 81803179), the China Postdoctoral Foundation (2021M692543), the Shaanxi Postdoctoral Foundation (2018BSHYDZZ47 and 2018BSHEDZZ96), the National Key R&D Program of China (2016YFE0119100), the Natural Science Foundation of Shaanxi Province (No. 2020JQ940), the Key R&D Plan of Shaanxi Province (No. 2021SF-025), and Scientific Research Projects of Xi'an Health Commission (No. 2021ms07).

## Conflict of Interest

The authors declare that the research was conducted in the absence of any commercial or financial relationships that could be construed as a potential conflict of interest.

## Publisher's Note

All claims expressed in this article are solely those of the authors and do not necessarily represent those of their affiliated organizations, or those of the publisher, the editors and the reviewers. Any product that may be evaluated in this article, or claim that may be made by its manufacturer, is not guaranteed or endorsed by the publisher.
